# Supplementary material for: Face classification using electronic synapses
Source: Nat Commun. 2017 May 12;8:15199. doi: 10.1038/ncomms15199 (PMC5437298; doi:10.1038/ncomms15199)
Supplement: Supplementary Information — Supplementary Figures and Supplementary Notes [file ncomms15199-s1.pdf]

Supplementary Figures

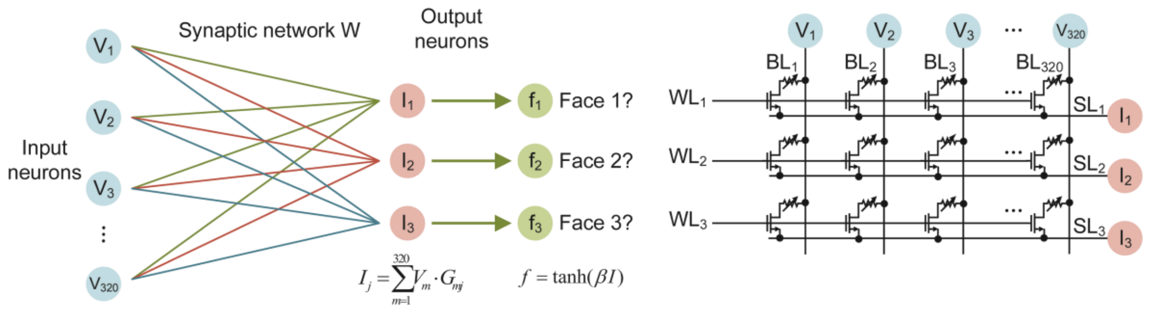

Supplementary Figure 1. The schematic of the perceptron. Here '*m*' is the index of a pixel of an input pattern and can be defined from 1 to 320, '*j*' represents the number of the output neuron and ranges from 1 to 3, matching the three categories.

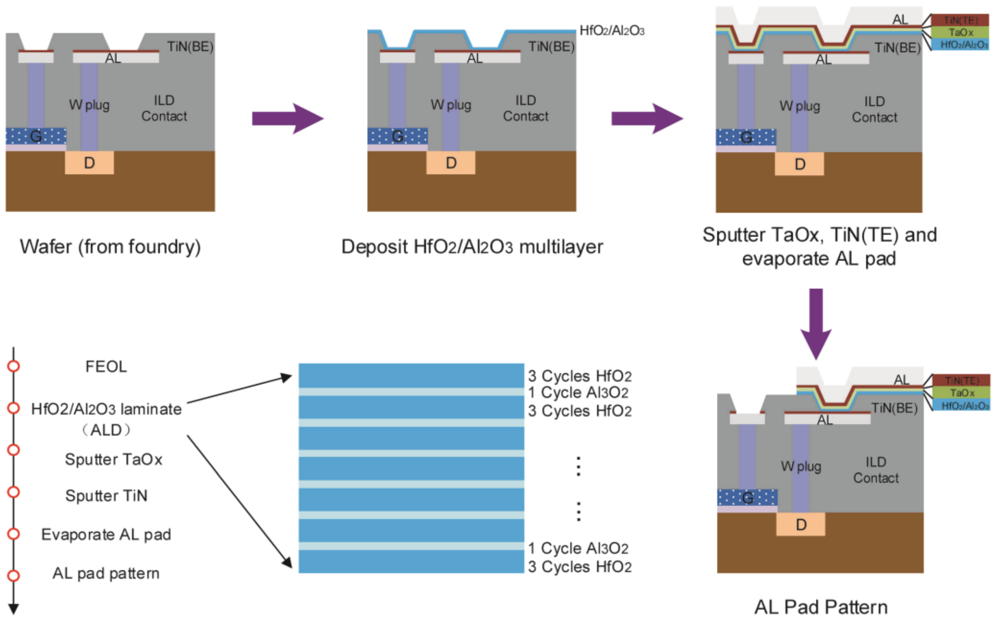

Supplementary Figure 2. Fabrication process for the RRAM stack.

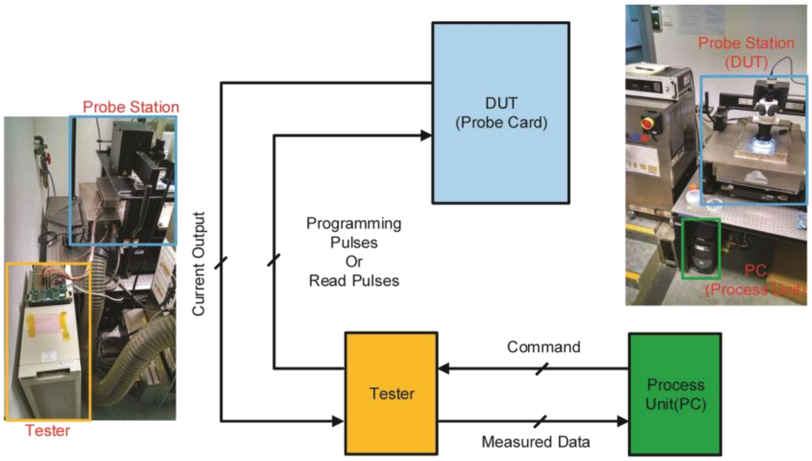

Supplementary Figure 3. The highly automatic test platform.

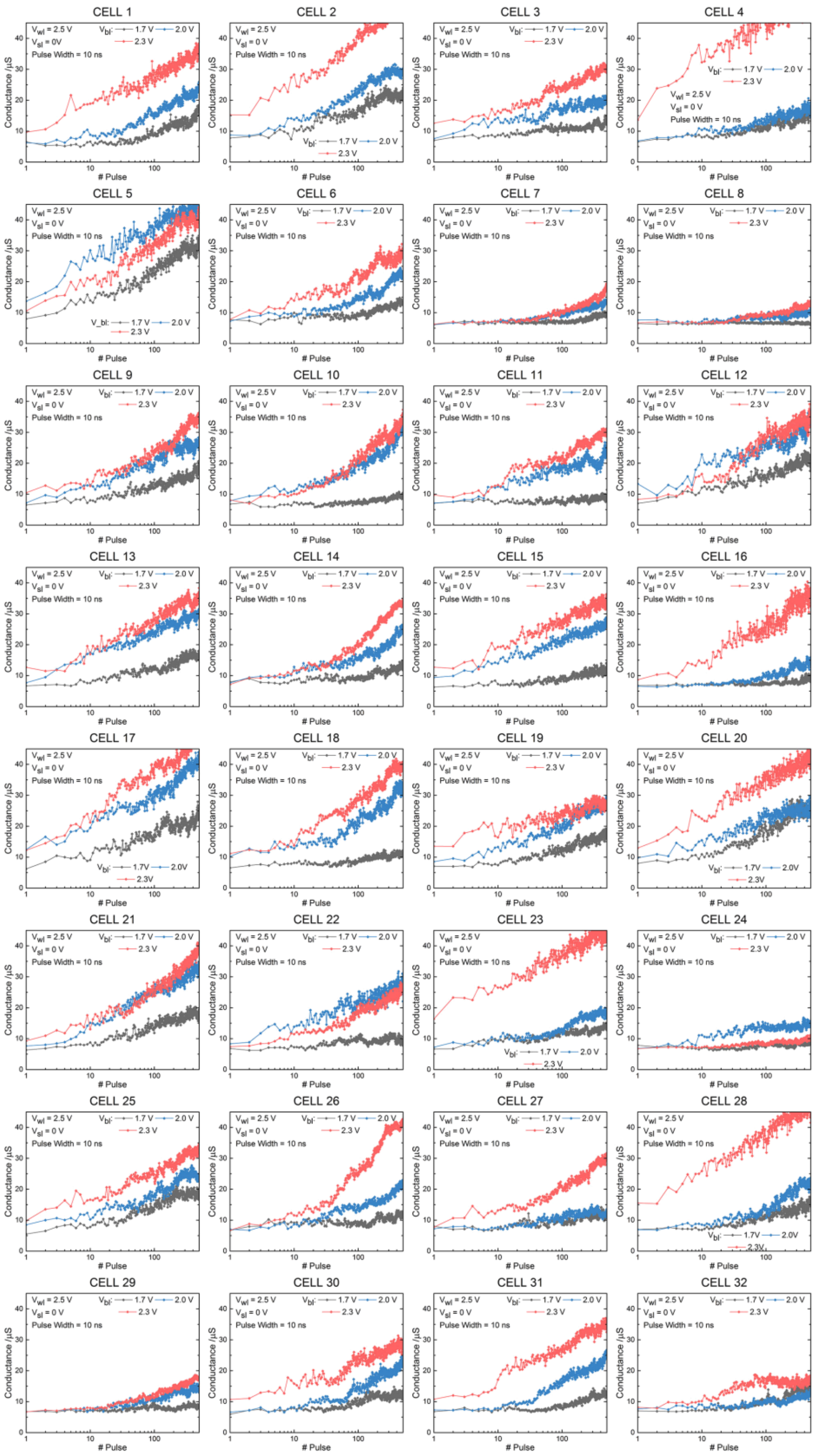

Supplementary Figure 4. (**a**) The SET process for 32 cells under identical pulse train with three different voltage amplitudes that V_{bl} = 1.7 V, 2.0 V, 2.3 V (V_{wl} = 2.5 V, pulse width = 10 ns).

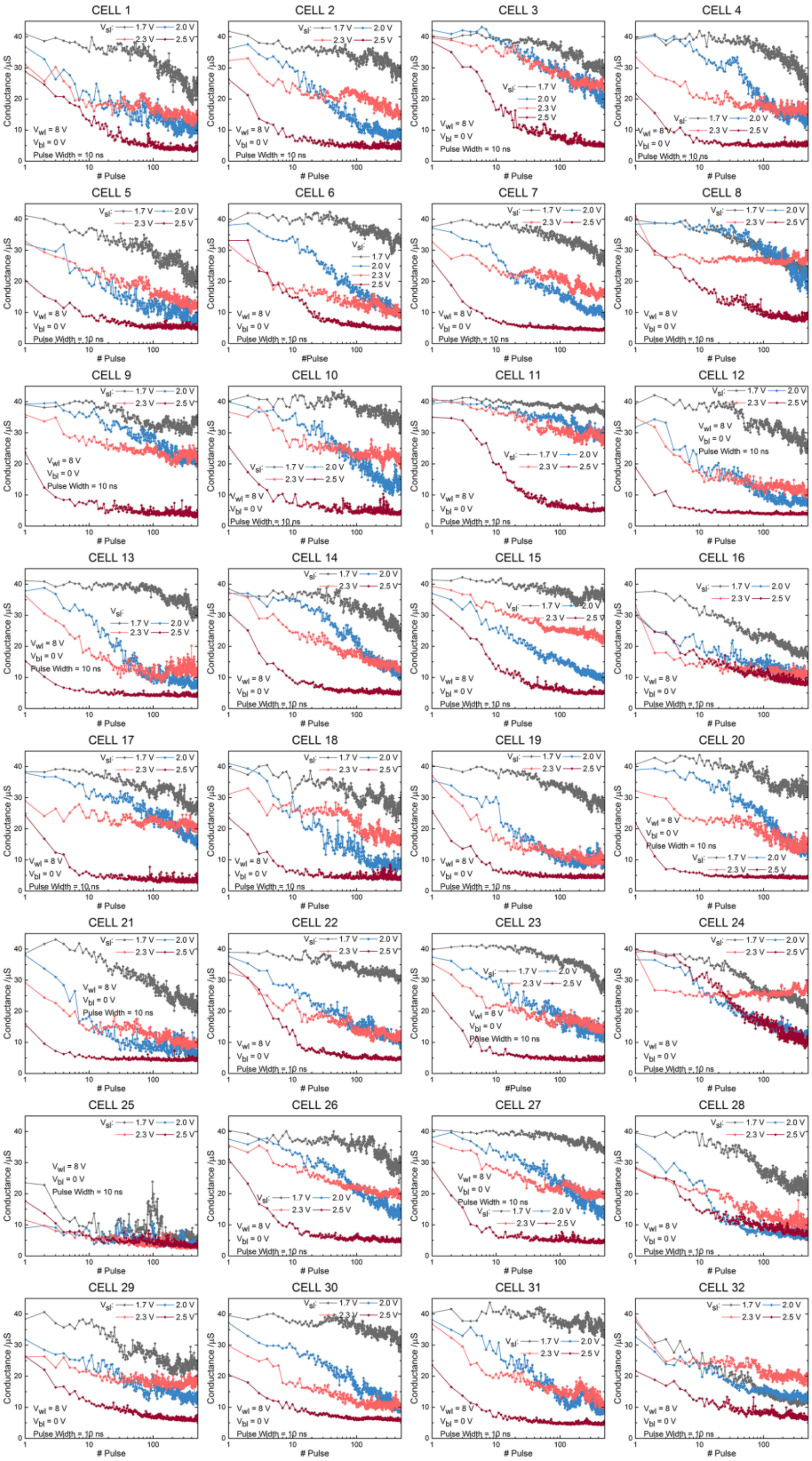

Supplementary Figure 4. (**b**) The RESET process for 32 cells under identical pulse train with four different voltage amplitudes that V_{sl} = 1.7 V, 2.0 V, 2.3 V and 2.5 V (V_{wl} = 8 V, pulse width = 10 ns).

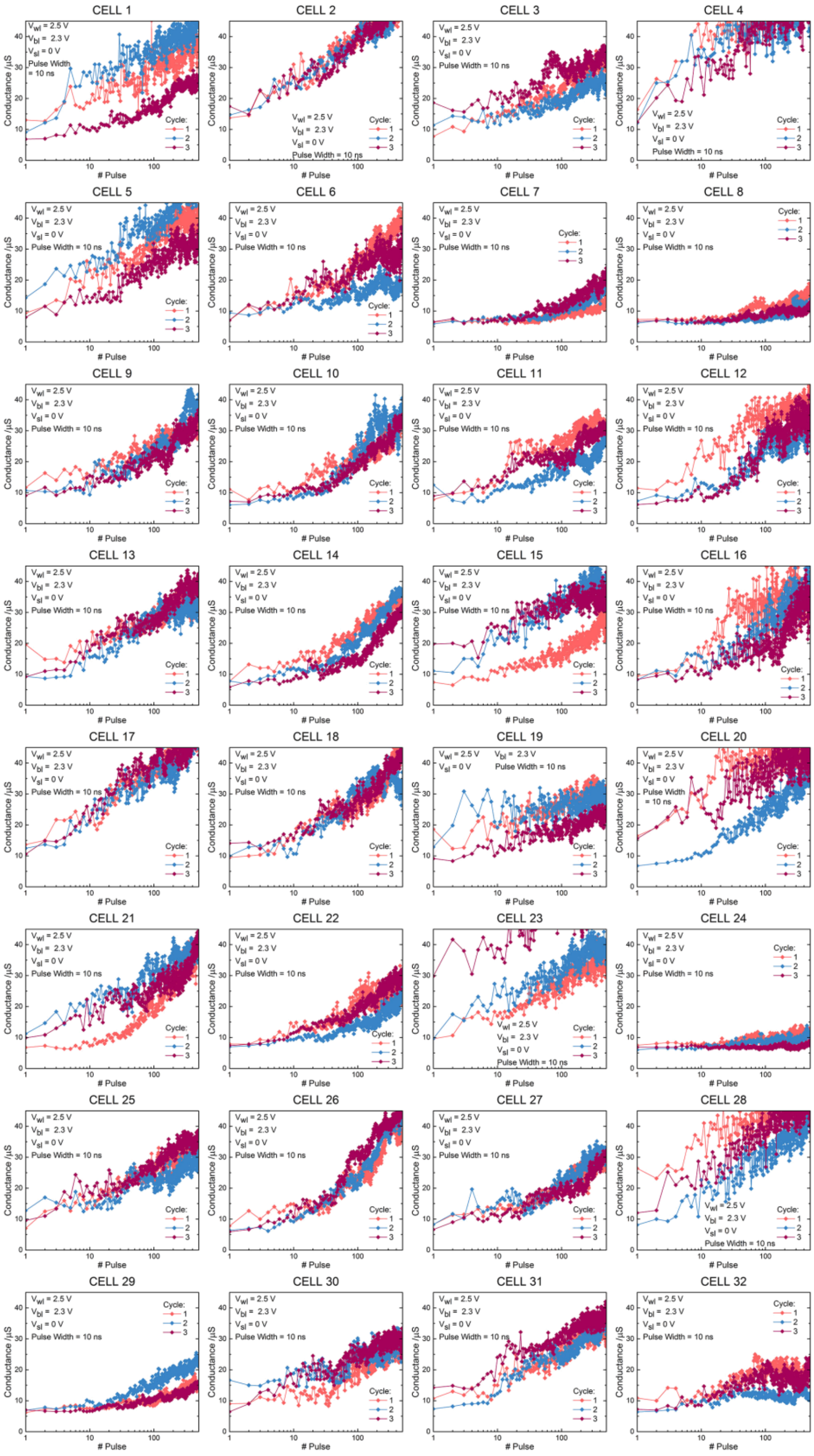

Supplementary Figure 5. (**a**) Three repeated SET cycles for 32 cells when V_{wl} = 2.5 V, V_{bl} = 2.3 V, V_{sl} = 0 V, pulse width = 10 ns.

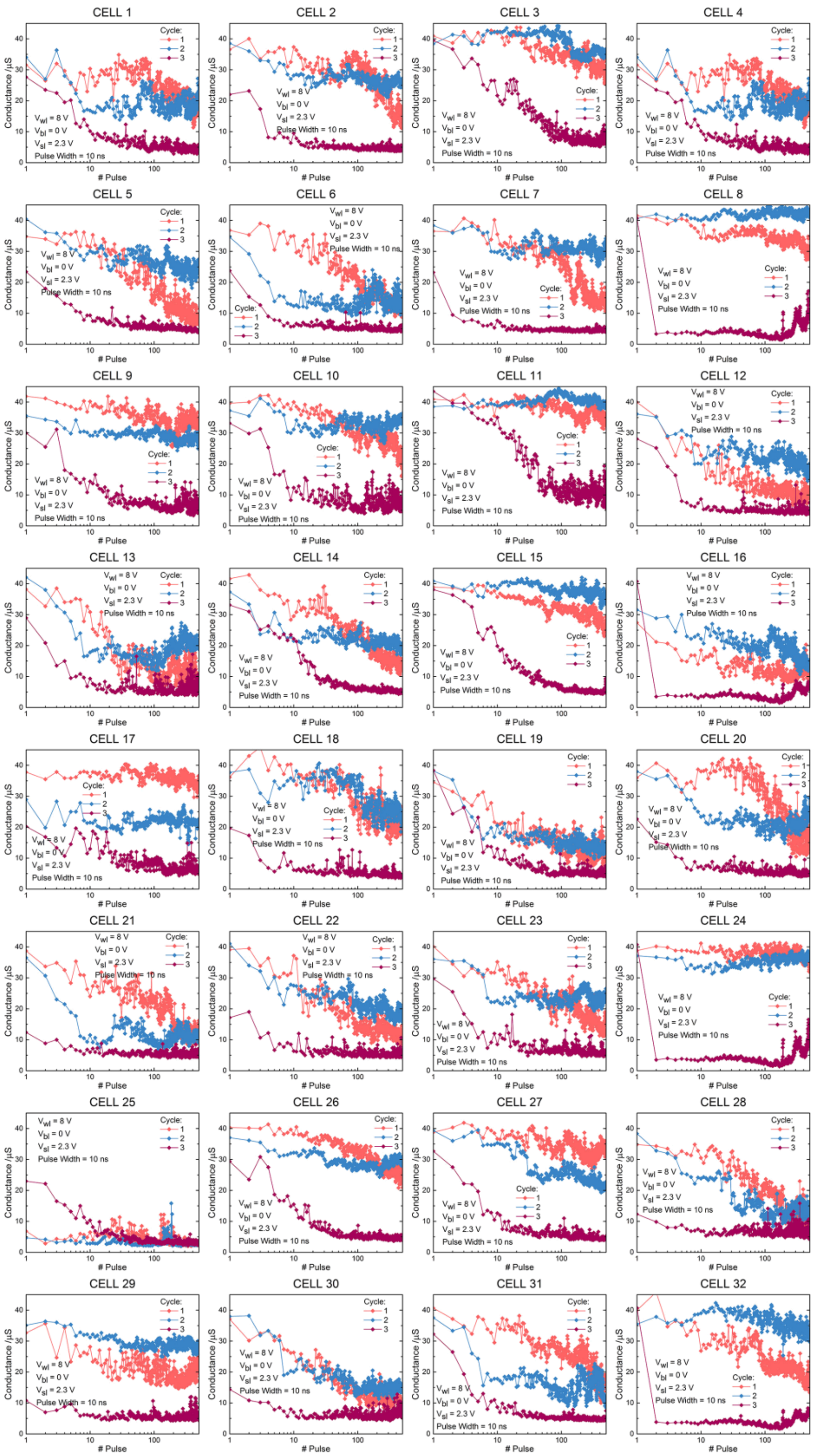

Supplementary Figure 5. (**b**) Three repeated RESET cycles for 32 cells when V_{wl} = 8 V, V_{bl} = 0 V, V_{sl} = 2.3 V, pulse width = 10 ns.

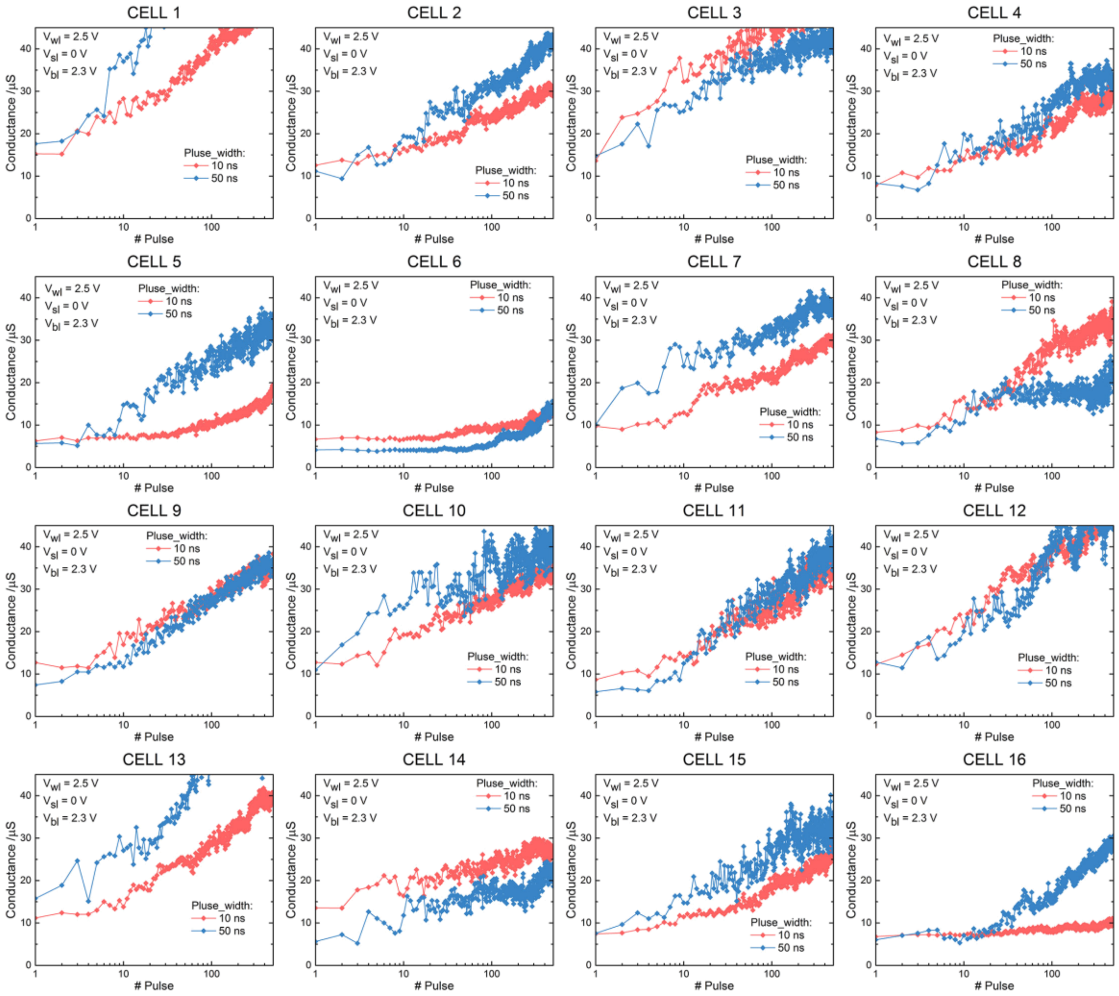

Supplementary Figure 6. (**a**) The comparison between 50 ns and 10 ns pulse widths on 16 1T1R cells during SET process when V_{wl} = 2.5 V, V_{bl} = 2.3 V, V_{sl} = 0 V.

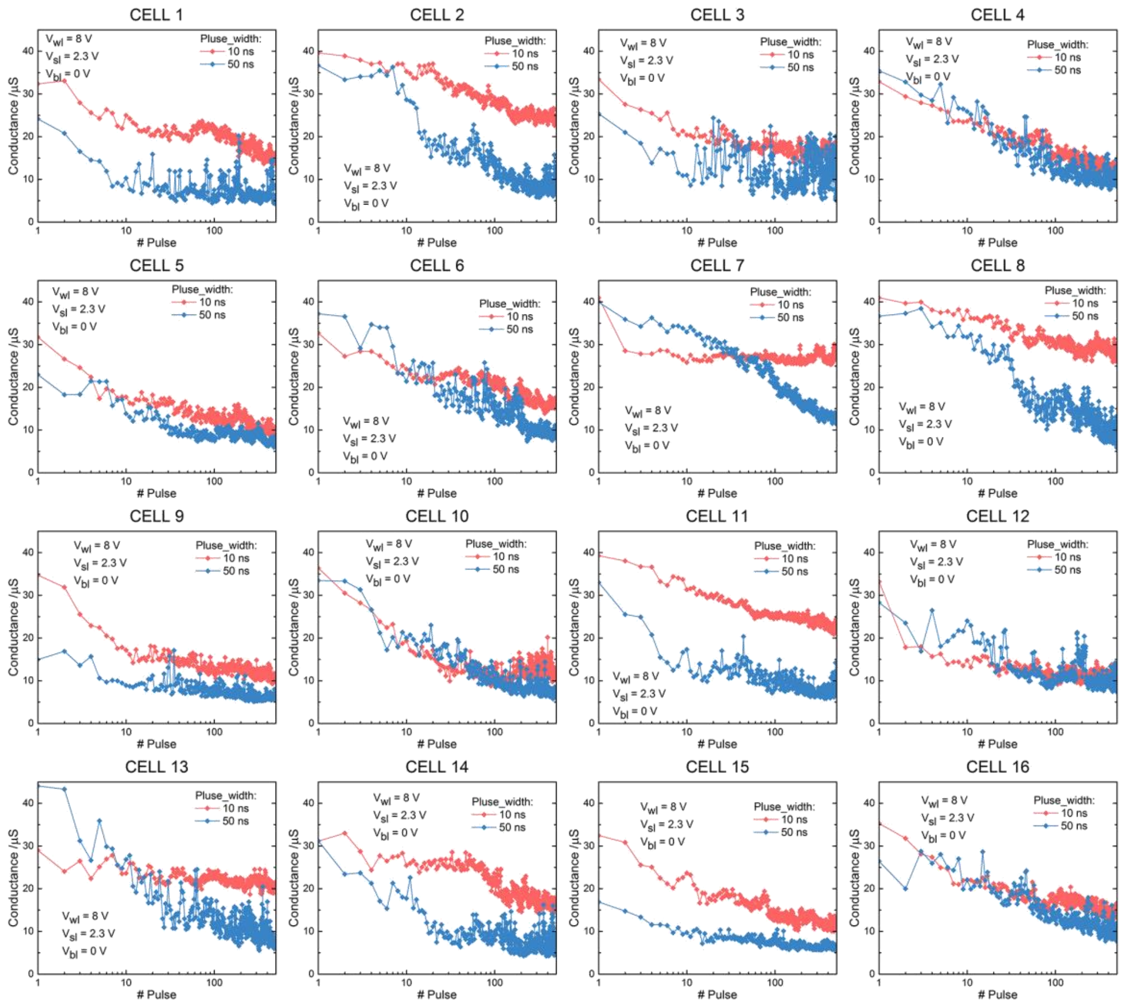

Supplementary Figure 6. (**b**) The comparison between 50 ns and 10 ns pulse widths on 16 1T1R cells during RESET process when V_{wl} = 8 V, V_{bl} = 0 V, V_{sl} = 2.3 V.

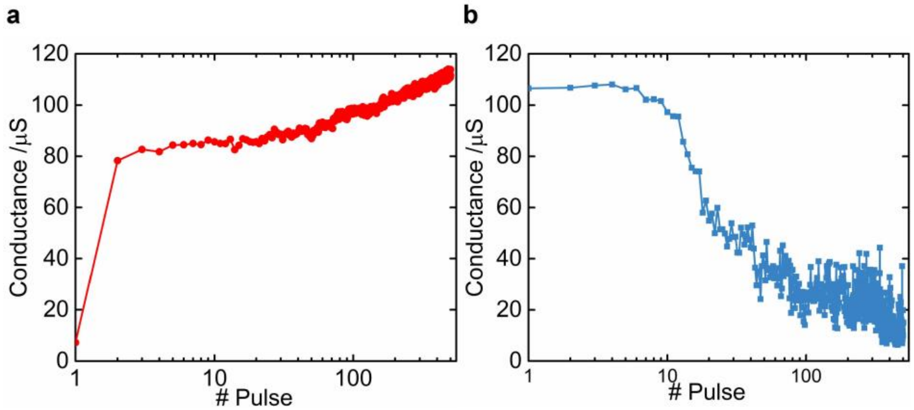

Supplementary Figure 7. An example of the typical bidirectional analog switching behavior of RRAM without HfO_x/AlO_y laminate structure. (**a**) Continuous conductance tuning performance under an identical pulse train condition during SET process. V_{wl} = 3.5 V, V_{bl} = 1.6 V / 50 ns, V_{sl} = 0 V. (**b**) Continuous conductance tuning performance during RESET operation. V_{wl} = 5 V, V_{bl} = 0 V, V_{sl} = 1.6 V / 50 ns.

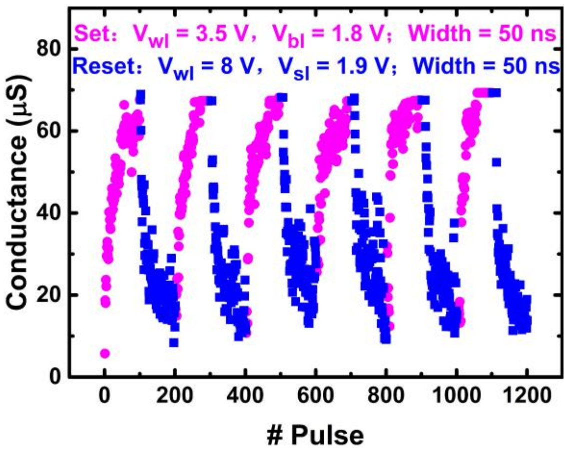

Supplementary Figure 8. The continuous conductance transferring under successive SET and RESET pulse cycles. It shows that the conductance can be modulated by applying identical voltage pulses.

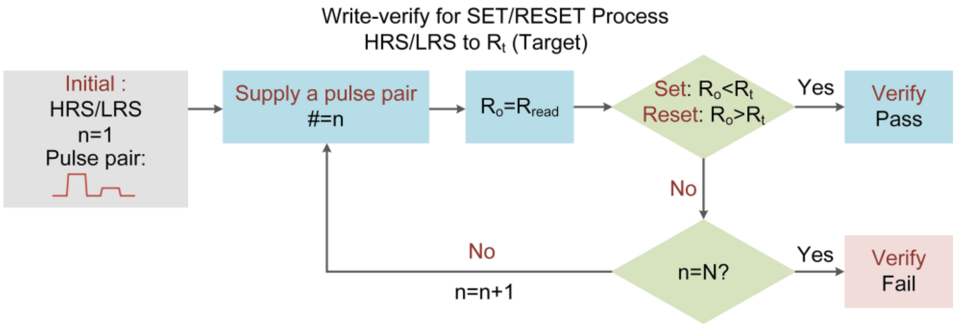

Supplementary Figure 9. The flow-chart of how write-verify works. N is set as the pulse number limitation, R_t is the target resistance state and R_o is the sensed resistance after each programming pulse.

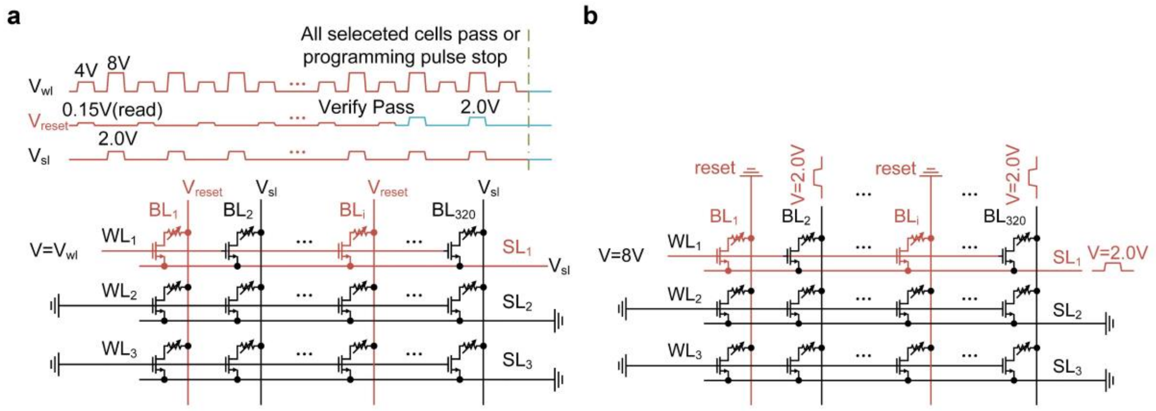

Supplementary Figure 10. An example of the RESET programming waveform applied on the first row to adjust the weight. (**a**) Waveforms for programming with write-verify. (**b**) Waveforms for programming without write-verify.

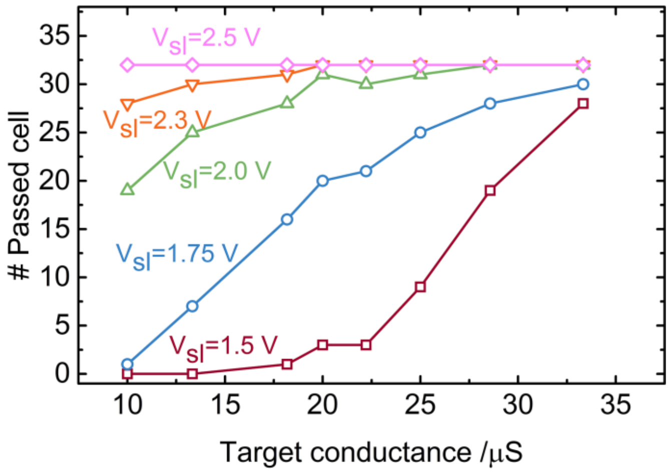

Supplementary Figure 11. The conductance modulation range measurement during RESET process with write-verify scheme under different pulse amplitudes. Y label represents the number of cells which are capable of reaching the target conductance within the limited 500 programming pulses.

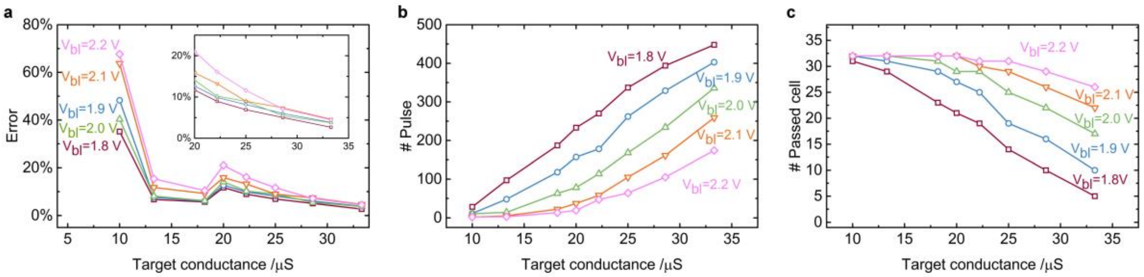

Supplementary Figure 12. Device performance during write-verify SET process. (**a**) The precision measurement result during SET process using verified pulse train with different amplitudes. (**b**) Y-axis represents the number of pulses needed to reach the target conductance from the same initial state 4 μS. These curves show the relationship of tuning speed with respected to different programming pulse amplitudes. (**c**) The conductance modulation range measurement during write-verify SET process under different pulse amplitudes.

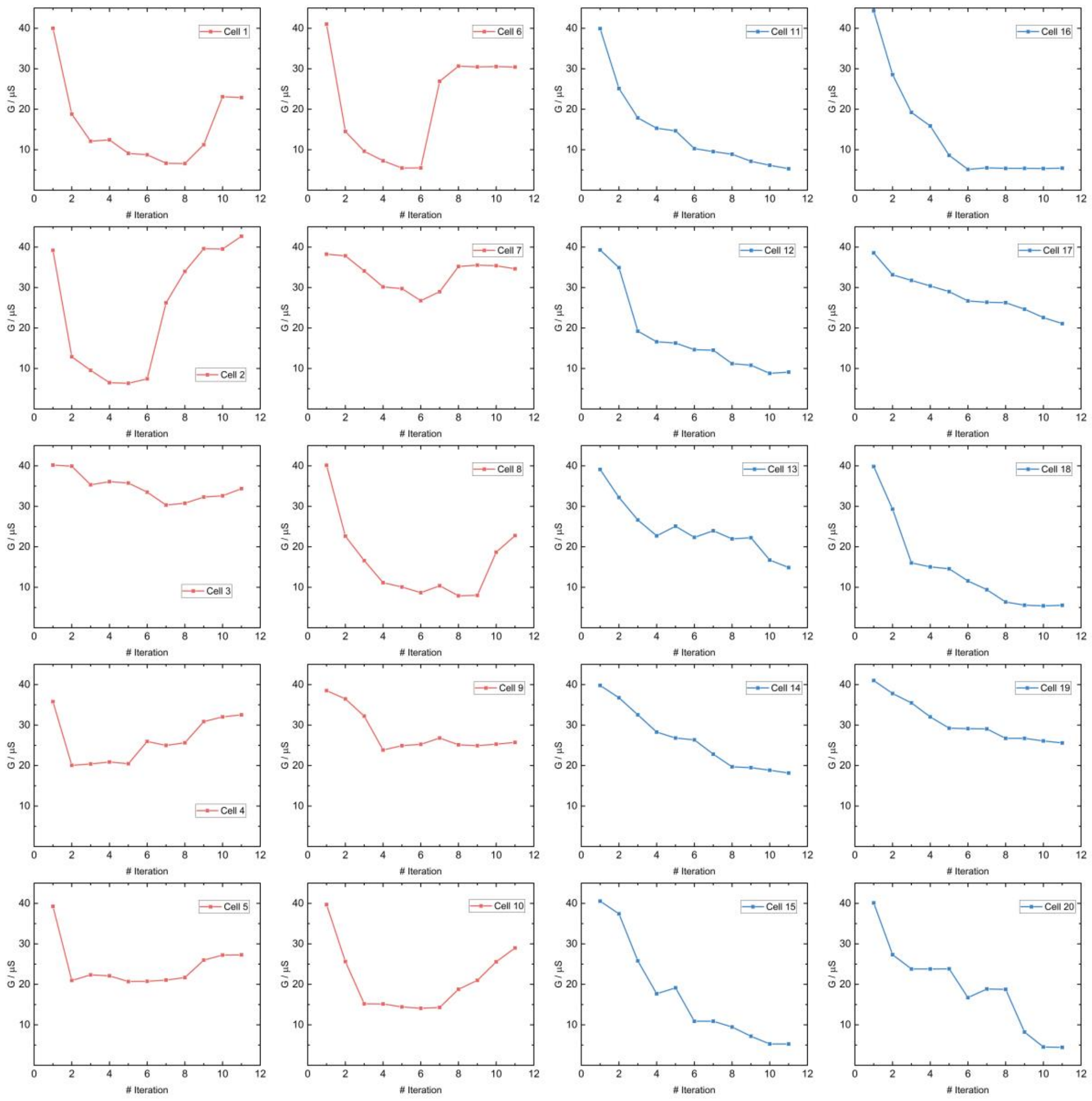

Supplementary Figure 13. Conductance evolution of 20 randomly selected RRAM devices during learning process under the write-verify scheme. The figures with red lines indicates the cells which experience SET processes. And the figures with blue lines indicate the cells which merely experience RESET processes.

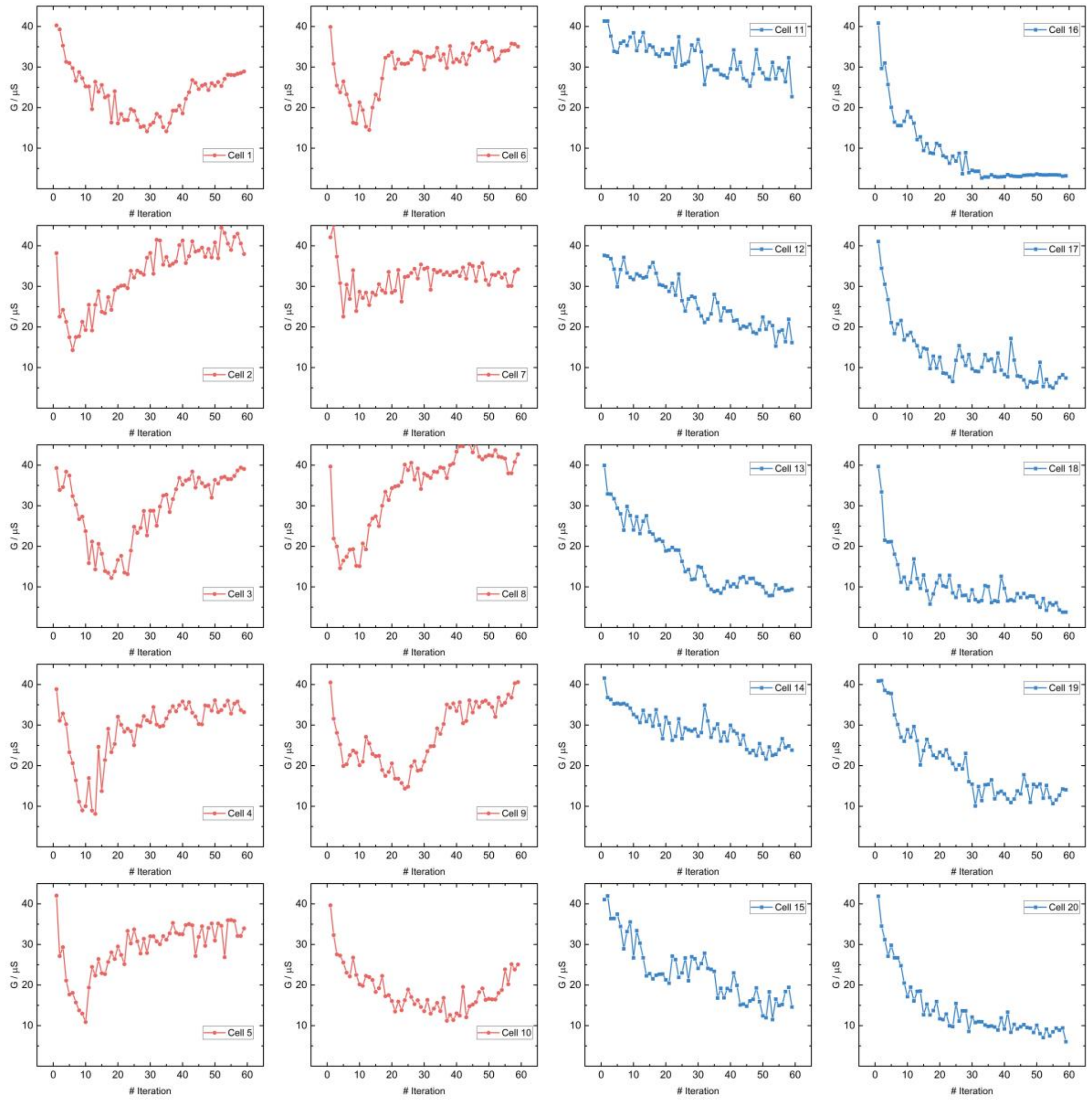

Supplementary Figure 14. Conductance evolution of 20 randomly selected RRAM devices during learning process under the without write-verify scheme. The figures with red lines indicates the cells which experience SET processes. And the figures with blue lines indicates the cells which merely experience RESET processes.

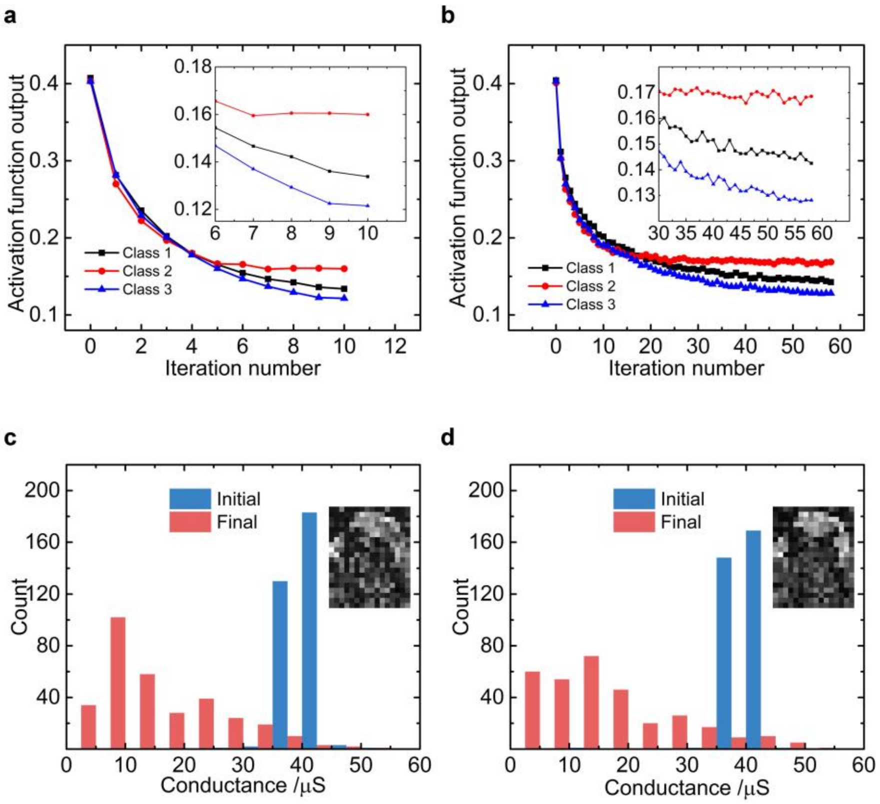

Supplementary Figure 15. The training process of the experimental demonstration referring to the 2nd class. (**a**) The activation function output value of the first class versus the iteration number using the write-verify scheme. The inset figure zooms in the several last steps. (**b**) The training process for programming without write-verify. (**c**) The initial and final conductance distribution comparison of the 2nd row when updating with write-verify. Inset shows the final conductance map. (**d**) The conductance distribution of the 2nd row and the conductance map for the case without write-verify. There are more cells locating in lower conductance range for the write-verify programming method and the energy consumption benefits from such a result.

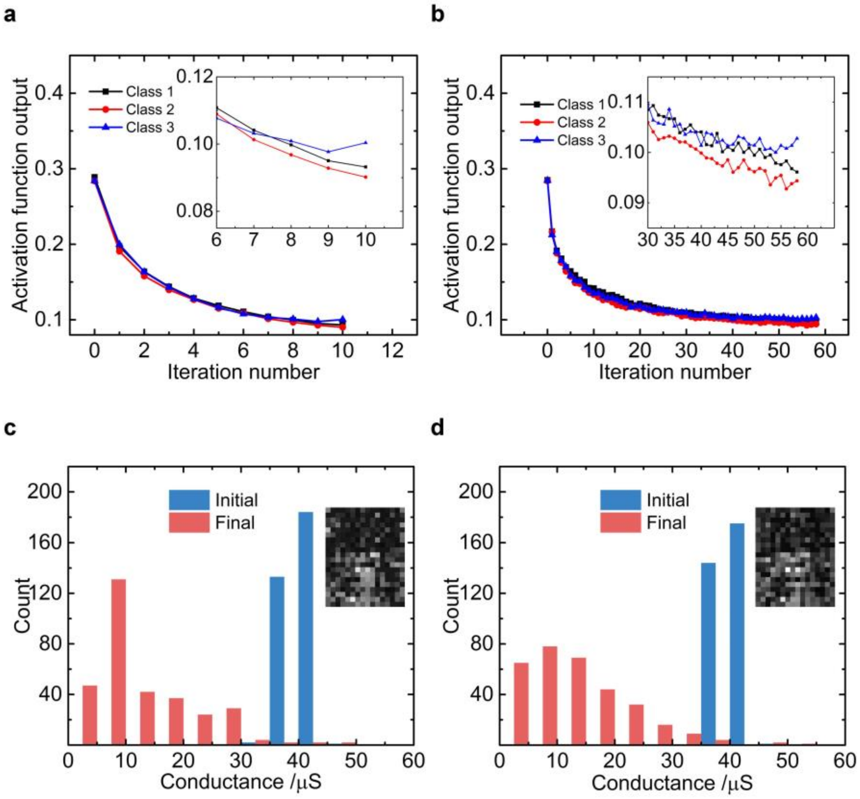

Supplementary Figure 16. The training process of the experimental demonstration referring to the 3rd class. (**a**) The activation function output value of the first class versus the iteration number using the write-verify scheme. The inset figure zooms in the several last steps. (**b**) The training process for programming without write-verify. (**c**) The initial and final conductance distribution comparison of the 3rd row when updating with write-verify. Inset shows the final conductance map. (**d**) The conductance distribution of the 3rd row and the conductance map for the case without write-verify. There are more cells locating in lower conductance range for the write-verify programming method and the energy consumption benefits from such a result.

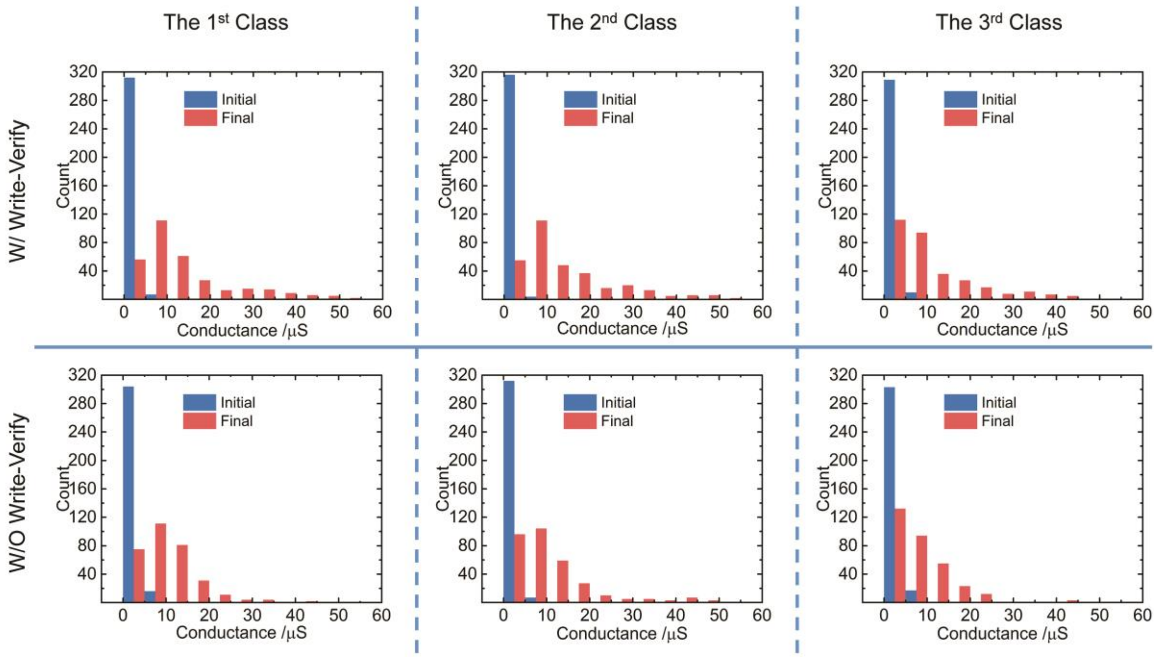

Supplementary Figure 17. The comparisons of initial and final conductance distribution under the proposed two updating schemes starting from the OFF state. The three figures above show the comparative distribution of 1st class, 2nd class and 3rd class under write-verify scheme, respectively. The three figures below show the comparative distribution of 1st class, 2nd class and 3rd class under without write-verify scheme, respectively.

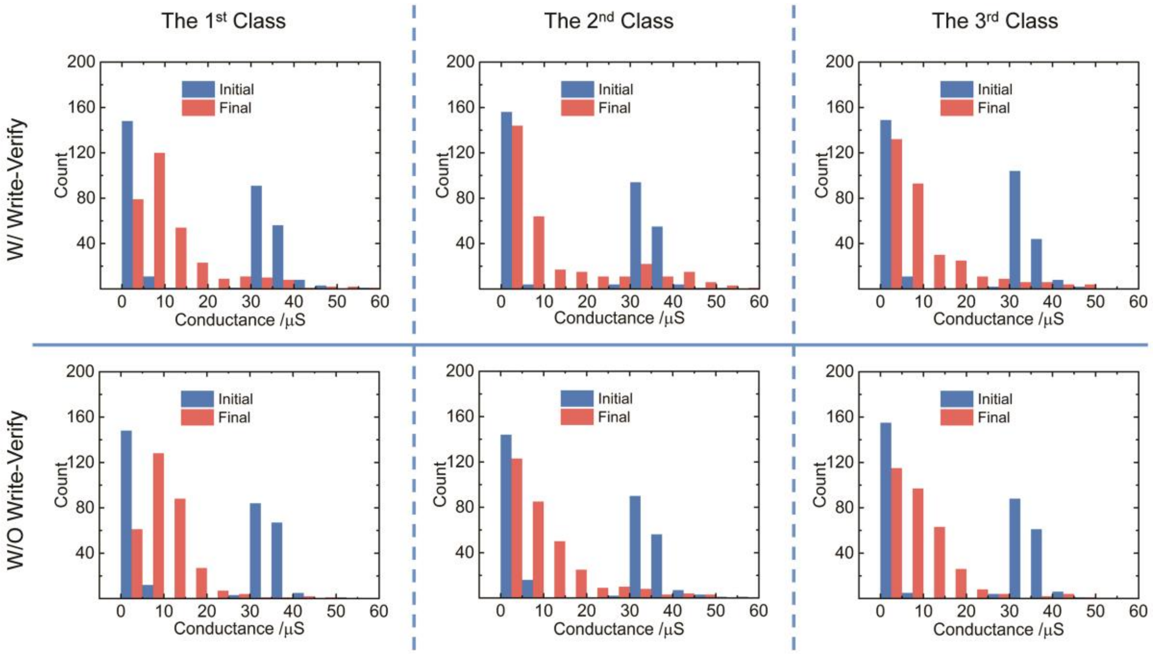

Supplementary Figure 18. The comparisons of initial and final conductance distribution under the proposed two updating schemes starting from a wide-distribution state. The three figures above show the comparative distribution of 1st class, 2nd class and 3rd class under write-verify scheme, respectively. The three figures below show the comparative distribution of 1st class, 2nd class and 3rd class under without write-verify scheme, respectively.

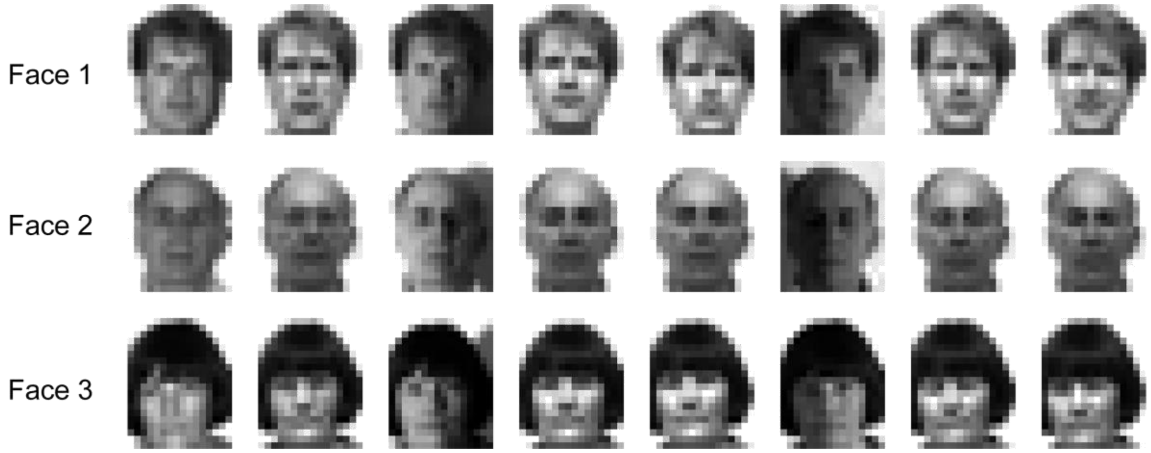

Supplementary Figure 19. The total 24 unseen test images from the Yale Face Database.

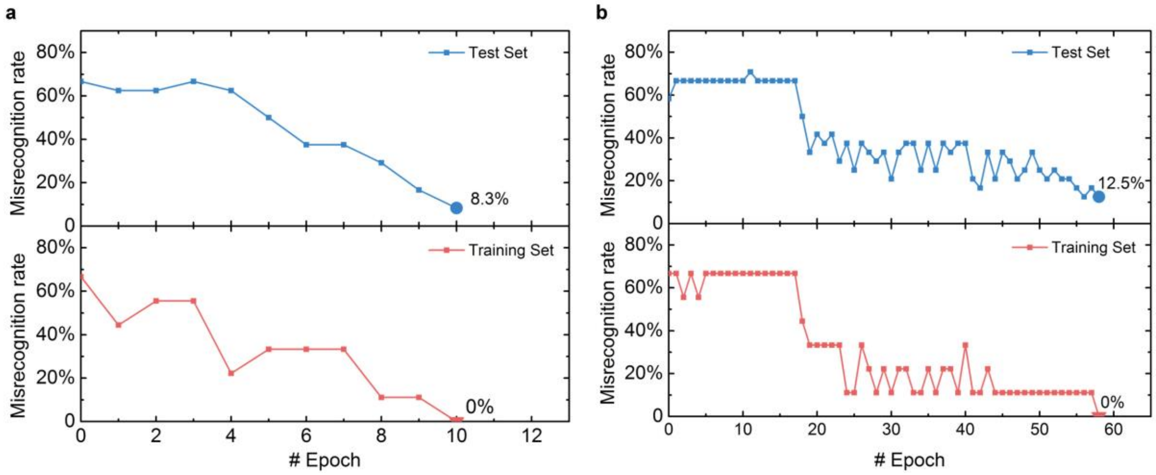

Supplementary Figure 20. Misrecognition rate after each epoch during training process. (**a**) The real-time changes of the misrecognition rate under scheme with write-verify. (**b**) The real-time changes of the misrecognition rate under scheme without write-verify.

Supplementary Notes

Supplementary Note 1

Bi-directional continuous conductance tuning performance at array level

After the optimization of the RRAM stacks, a 1024-cell-1T1R array with 128 rows and 8 columns is deposited as shown in Fig. 1b of the main text. This 1T1R array has some remarkable characteristics, such as high operation speed around 10 ns and high bit yield (99.99%), robust endurance performance and a stable switching window ranging from 25 kΩ to 250 kΩ under appropriate bidirectional operating pulse voltage (2 V / 50 ns), leading to a relatively low programming energy consumption. Further, the bi-directional analog conductance tuning behavior is generally captured in this integrated array and the performance of 32 randomly chosen cells are shown below. The conductance is sensed after each programming pulse.

Each figure stands for an individual cell and each curve represents the conductance continuous tuning performance under a certain identical pulse train. The pulse width is set at 10 ns. Considering cycle-to-cycle fluctuation, the raw data is analyzed at each certain pulse condition by statistically averaging over 3 repeated procedures. Supplementary Fig. 4a (SET) and Supplementary Fig. 4b (RESET) show the inherent device-to-device variance and how the pulse amplitude affects the bi-directional analog behavior. The curve trend implies that the larger pulse amplitude is, the wider tuning range it achieves. A larger pulse amplitude also results in higher changing step for both SET and RESET process. The bi-directional analog switching performance is generally realized while the device-to-device variation exists. Whatever the pulse amplitude is, the initial state is 6.67 μS (150 kΩ) for SET process and 40 μS (25 kΩ) for RESET process for every 1T1R cell.

To evaluate the influence of cycle-to-cycle variance, three repeated procedures are conducted on 32 randomly chosen cells, just as Supplementary Fig. 5a (SET) and Supplementary Fig. 5b (RESET) illustrate. The start state is set to the same with Supplementary Fig. 4. The pulse condition is specified in the plot. The fluctuation is inevitable.

Besides some tests are carried out to see the impact of the different pulse widths on 16 randomly chosen cells. 50 ns pulse width and 10 ns pulse width are employed. The voltage is the same with that of Supplementary Fig. 5. Just as Supplementary Fig. 6a (SET) and Supplementary Fig. 6b (RESET) prove, the tuning speed is faster, the tuning range is wider while the tuning accuracy is lower for 50 ns pulse width. Considering all these above, the pulse condition must be chosen carefully.

Supplementary Note 2

Bi-directional continuous conductance tuning performance of RRAM without laminate structure

To improve the bidirectional analog switching performance, the HfO_x/AlO_y laminate structure is leveraged to control the generation of oxygen vacancies in $TiN/TaO_x/HfAl_yO_x/TiN$ stack structure. Supplementary Fig. 7 shows an example of the

typical continuous conductance tuning performance of a RRAM cell without HfO_x/AlO_y laminate structure, i.e. $TiN/TaO_x/HfO_2/TiN$ stacks, under an identical pulse train condition during SET and RESET process. Compared with Fig. 3b and Fig. 3c in the main text, this structure without optimization presents a greater changing step regardless of whether the conductance is increasing or decreasing.

Supplementary Note 3

Conductance evolution trace during training iteration

During the training process of the experimental demonstration, there are 19.3% of the devices experience SET transition under the write-verify scheme and 14.6% of the devices experience SET transition under the without write-verify scheme. 20 RRAM devices under the two proposed programming schemes are selected to show their conductance evolution trace in Supplementary Fig. 13 and Supplementary Fig. 14. Half of the 20 devices experience SET transitions and the other merely experience RESET transitions.

Supplementary Note 4

The system converges from different initial conductance distribution states

In the main text, the perceptron is trained from a tight high-conductance distribution around 40 μS. Furthermore, another two demonstrations are carried out, one starting from a tight low conductance distribution around 4 μS and another proceeding from a wide conductance distribution state. Since the device has bi-directional analog switching behavior, it does not matter what the initial conductance distribution is and both succeed to converge. The initial and final conductance distribution comparison are presented in Supplementary Fig. 17 and Supplementary Fig. 18.

Supplementary Note 5

Recognition rate on Yale Face Database during training process

The test process convinces the generalization ability of the perceptron by employing a test image set. Supplementary Fig. 20 shows the generalization performance of such a neuromorphic network, i.e. the real time change of misrecognition rate when identifying the training images and test images during training process. The conductance weights are recorded after each iteration and used to compute misrecognition rate by computer.

Supplementary Note 6

Estimation of Intel Xeon Phi hardware for comparison

We pay attention to the energy consumption on operation of the 1T1R array which includes the multiply operation and weight updating process. For a fair comparison with the same task implemented in this work, we estimate the energy consumption of the same operations within Intel Xeon Phi processor with off-chip storage as well as Intel Xeon Phi processor with on-chip integrated RRAM. The energy for the operations beyond the multiply operation and weight updating process is not taken into consideration for

comparison, such as activation function tanh, transferring the input image data, aggregating and storing the weight updates during batch-based programming.

The task implemented within analog RRAM in the experiment reported in this paper is equivalent to these tasks: 1) Reading the synaptic weights, 2) Vector-matrix multiplication of synaptic weights with input images 3) Updating the synaptic weights 4) Writing back the synaptic weights. Estimation of these tasks on Intel Xeon Phi is done by using the energy model of Intel Xeon Phi processor reported in *(36)* in the main text. According to *(36)*, a register-to-register vector operation with 512 bit wide registers consume ~ 1 nJ. We assume 16 bit synapses, which makes a vector operation an operation on 32 numbers each of which are 16 bits. Tasks 2 and 3 above are done within the processor: task 2 is equivalent to 60 vector operations for each image within an epoch, corresponding to 540 vector operations for 9 images within 1 epoch. Task 3 corresponds to the sum of two weight matrices, which in Intel Xeon Phi is equivalent to 30 vector sums; consuming 30 nJ. Hence, task 2 and 3 consume 570 nJ and use processor and registers only. Tasks 1 and 4 involve memory/storage access. Since the update can be expected to be performed relatively less often in a real life scenario, the weights can be expected to stay on an off-chip storage. In case of NAND, off-chip storage access for 2 KB page (around the same as the size of weight matrix in our case) consumes ~ 38 μJ, which dominates all other numbers estimated above. When the storage is assumed to be an on-chip monolithically integrated RRAM and when only the energy within digital RRAM is taken into account (not the wires, periphery, etc), task 1 and 4 consume 0.4 nJ and 132 nJ, respectively. Task 4 is estimated as follows:

$$\text{Energy} = 320 \times 3 \times 16\ \text{bits} \times (2.8\ \text{V})^2 \times G_{\text{average}} \times 50\ \text{ns}$$

where 320×3 is the size of weight matrix, G_{average} is the mean of LRS and HRS conductance values (25 kΩ and 250 kΩ, respectively). Then energy consumption is 132 nJ. Energy for task 1 is estimated similarly, except that instead of 2.8 V pulse, 0.15 V reading voltage is used.
